# Clinical and Multimodal Imaging Findings and Risk Factors for Ocular Involvement in a Presumed Waterborne Toxoplasmosis Outbreak, Brazil^1^

**DOI:** 10.3201/eid2612.200227

**Published:** 2020-12

**Authors:** Camilo Brandão-de-Resende, Helena Hollanda Santos, Angel Alessio Rojas Lagos, Camila Munayert Lara, Jacqueline Souza Dutra Arruda, Ana Paula Maia Peixoto Marino, Lis Ribeiro do Valle Antonelli, Ricardo Tostes Gazzinelli, Ricardo Wagner de Almeida Vitor, Daniel Vitor Vasconcelos-Santos

**Affiliations:** Universidade Federal de Minas Gerais, Belo Horizonte, Brazil (C. Brandão-de-Resende, H.H. Santos, A.A.R. Lagos, C.M. Lara, J.S.D. Arruda, R.W.A, Vitor, D.V. Vasconcelos-Santos);; Centro de Pesquisas René Rachou, Fundação Oswaldo Cruz, Belo Horizonte (A.P.M.P. Marino, L.R.V. Antonelli, R.T. Gazzinelli).

**Keywords:** uveitis, posterior uveitis, toxoplasmosis, ocular toxoplasmosis, disease outbreaks, multimodal imaging, optical coherence tomography, parasites, waterborne diseases, zoonoses, Brazil

## Abstract

In 2015, an outbreak of presumed waterborne toxoplasmosis occurred in Gouveia, Brazil. We conducted a 3-year prospective study on a cohort of 52 patients from this outbreak, collected clinical and multimodal imaging findings, and determined risk factors for ocular involvement. At baseline examination, 12 (23%) patients had retinochoroiditis; 4 patients had bilateral and 2 had macular lesions. Multimodal imaging revealed 2 distinct retinochoroiditis patterns: necrotizing focal retinochoroiditis and punctate retinochoroiditis. Older age, worse visual acuity, self-reported recent reduction of visual acuity, and presence of floaters were associated with retinochoroiditis. Among patients, persons >40 years of age had 5 times the risk for ocular involvement. Five patients had recurrences during follow-up, a rate of 22% per person-year. Recurrences were associated with binocular involvement. Two patients had late ocular involvement that occurred >34 months after initial diagnosis. Patients with acquired toxoplasmosis should have long-term ophthalmic follow-up, regardless of initial ocular involvement.

## Abstract 2

After a 2015 outbreak, 23% of patients had retinochoroiditis, indicating that patients with acquired toxoplasmosis should have long-term follow-up, regardless of initial ocular involvement.

Toxoplasmosis is caused by *Toxoplasma gondii*, an obligate intracellular apicomplexan parasite that infects up to one third of the human population (*1*–*4*). Humans are mainly infected by ingesting tissue cysts in undercooked or raw meat or oocysts excreted in cat feces that contaminate water or food (*1*–*4*). Ocular disease is the major clinical repercussion in immunocompetent patients; toxoplasmosis is the leading cause of infectious posterior uveitis worldwide and can potentially lead to severe ocular complications (*1*,*3*,*5*,*6*). Although congenital toxoplasmosis more frequently leads to retinochoroiditis, postnatally acquired infection now is acknowledged as being associated with a large proportion of cases (*2*,*3*,*6*,*7*). Association between ocular toxoplasmosis and older age is not completely understood (*8*), but previous studies found increased prevalence of ocular involvement in persons >30 years of age (*9*,*10*).

Toxoplasmosis outbreaks are good opportunities to clarify clinical aspects of this complex disease because patients are infected at known times, by similar routes, and presumably by parasites of the same genotype (*11*–*19*). In 2015, an outbreak of presumed waterborne toxoplasmosis was reported in Gouveia, a small city of »10,000 inhabitants in the center of the state of Minas Gerais in southeastern Brazil (*20*). Municipal, state, and federal health authorities investigated several cases of fever, malaise, weight loss, and lymphadenopathy. Recent toxoplasmic infection was eventually confirmed in 52 cases. All patients had the disease after drinking water from a single, presumably contaminated, source (*20*).

We performed complete ophthalmic examination and multimodal fundus imaging evaluation on all 52 patients. The objective of this study was to describe clinical and multimodal imaging findings and determine the prevalence of ocular involvement, incidence of recurrences and complications, and to analyze risk factors for ocular involvement in this cohort.

## Methods

We used a prospective cohort approach to address our main goal. The study was approved by institutional review boards of René Rachou Research Center, Oswaldo Cruz Foundation (CAAE no. 37614314.7.3001.5091), and Federal University of Minas Gerais (CAAE no. 37614314.7.3001.5149). All patients provided written informed consent.

We defined a case as illness in a person in the city of Gouveia with a history of fever, headache, lymphadenopathy, asthenia, or myalgia during February 12–May 18, 2015. From 5,276 local health charts, a task force comprised of municipal, state, and federal health authorities identified 201 persons suspected of meeting case definition criteria. Before confirmation of toxoplasmosis, differential diagnosis was made with consultation of an infectious disease specialist and serologic tests for dengue fever, visceral leishmaniasis, and leptospirosis. 

We contacted the 201 persons with suspected toxoplasmosis. We were able to reach 151 (75.1%) persons whom we subsequently interviewed and tested for toxoplasmosis. We defined confirmed cases of acute toxoplasmosis as persons having *T. gondii* IgM and low avidity IgG on enzyme-linked fluorescence assay by using Vidas Toxo IgM, IgGII, and IgG avidity assays (bioMérieux, https://www.biomerieux.com).

Among the 151 suspected cases interviewed and tested, 52 (34.4%) had serologic evidence of *T. gondii* IgM and low avidity IgG, indicating acute toxoplasmosis. We performed a complete ophthalmic examination on each of the 52 case-patients, including assessment of best-corrected visual acuity (VA), applanation tonometry, slit-lamp examination (SLE; biomicroscopy), and indirect ophthalmoscopy. All case-patients also underwent multimodal imaging evaluation, including fundus photography, reflectances, fundus autofluorescence, and spectral-domain optical coherence tomography (SD-OCT). For patients with confirmed ocular involvement, we also performed fluorescein angiography. We determined prevalence and incidence of ocular changes and clinical characteristics on the basis of clinical and multimodal imaging findings. All patients with ocular involvement received standard therapy for 35–45 days, which consisted of sulfadiazine (1 g 4×/d), pyrimethamine (25–50 mg/d), folinic acid (7.5 mg/d), and prednisone (40–60 mg/d). One patient was allergic to sulfa and was switched from sulfadiazine to clindamycin 300 mg 4×/d. Complete blood counts were monitored at baseline and every 2 weeks during treatment.

For 3 years, we conducted follow-up examinations on case-patients at 5–8-month intervals and conducted the same ophthalmic examination protocol periodically. We reexamined case-patients with active primary or recurrent retinochoroiditis 3–6 weeks after therapy, or more often, if needed. We defined severe ocular involvement as binocular or macular involvement, or extensive necrotizing retinochoroiditis of >3 disk diameter (DD).

We prospectively collected and stored in an electronic database clinical and ophthalmological data, including symptoms, best-corrected VA, applanation tonometry, SLE, indirect ophthalmoscopy, and multimodal imaging. We assessed best-corrected VA by using an early treatment of diabetic retinopathy study chart and reported results in logarithm of the minimum angle of resolution (logMAR) scale. Previous studies suggested increased prevalence of ocular involvement among patients >30 years of age and among patients >50 years of age (*9*,*10*). We also analyzed age >40 years at time of infection as a potential risk factor for occurrence and severity of ocular involvement.

We defined the time of primary infection for each case-patient as the time of ocular or systemic symptom onset. To calculate time intervals among persons with new active retinochoroidal lesions during follow-up, we assumed eye disease occurred when patients first noted ocular symptoms. For case-patients without ocular symptoms, we assumed eye disease occurred when consistent retinochoroidal lesions were identified. Among case-patients who displayed new retinochoroidal scars during follow-up exams, we assumed eye disease occurred when ocular symptoms first were perceived; if the case-patient did not notice any ocular symptoms, we assumed eye disease occurred in the time between the prior ocular examination and identification of the scar. We also noted the first instance of retinochoroidal recurrences in either eye.

We performed statistical analyses by using R version 3.5.2 (*21*) by nonparametric methods and considered p<0.05 statistically significant. We used Mann-Whitney-Wilcoxon test to compare continuous variables, including age, length of follow-up, and VA at baseline. We used the mid-p exact test to compare proportions between subgroups, such as age >40 years, sex, and presence of underlying conditions and symptoms. We reported continuous variables as median (interquartile range [IQR]) and proportions as no. (%). We estimated the 95% CI of relative risks by using a maximum-likelihood estimator and described the follow-up by using the rate of recurrence per person-year (*22*), rate of recurrence per person-month, and Kaplan-Meier survival plot (*23*). We estimated survival probability and cumulative risk for ocular involvement and ocular recurrence by using the Kaplan-Meier method and compared results by using a log-rank test (*24*,*25*).

## Results

### Baseline Examination

All 52 patients with serologic evidence of acute toxoplasmosis underwent a baseline examination in the first 4 months after onset of systemic symptoms; 40 (77%) patients had a baseline exam within the first month (Table 1). Median age at infection was 34 years (IQR 27–40 years); 8 (15.4%) patients were female and 44 (84.6%) were male. The most common systemic signs or symptoms were fever (52/52; 100%), headache (33/52; 63%), myalgia (30/52; 58%), and lymphadenopathy (8/52; 15%). Ocular symptoms were reported by 17 (32.6%) patients, among whom 9 (52.9%) reported recent VA decrease, 8 reported eye pain (47%), and 3 (17.6%) reported floaters.

**Table 1:** Characteristics and ocular signs and symptoms among patients with confirmed acute toxoplasmosis infection at baseline examination, Brazil*

Characteristics	Total, n = 52	No ocular involvement, n = 40	Ocular involvement, n = 12	p value
Age at infection, y, median (IQR)†	34 (27–40)	32 (22–38)	43 (40–47)	<0.01
>40 years of age†	14 (27)	6 (15)	9 (75)	<0.01
Sex				
M	44 (84.6)	33 (82.5)	11 (91.7)	0.50
F	8 (15.4)	7 (17.5)	1 (8.3)	0.50
Follow-up length, mo, median (IQR)	36 (19–36)	36 (12–36)	35 (24–37)	0.32
Underlying conditions				
Arterial hypertension	7 (13.5)	4 (10.0)	3 (25.0)	0.23
Diabetes mellitus	1 (1.9)	0	1 (8.3)	0.23
General signs and symptoms				
Fever	49 (94.2)	38 (95.0)	11 (91.7)	0.68
Headache	33 (63.5)	25 (62.5)	8 (66.7)	0.81
Myalgia	30 (57.7)	22 (55.0)	8 (66.7)	0.50
Lymphadenopathy	8 (15.4)	7 (17.5)	1 (8.3)	0.50
Ocular signs and symptoms				
VA at baseline, logMAR, median (IQR)†‡	0.0 (0.0–0.0)	0.0 (0.0–0.0)	0.1 (0.0–0.6)	0.01
Eye pain	8 (15.4)	5 (12.5)	3 (25.0)	0.33
Self-reported recent reduction of VA†	10 (19.2)	1 (2.5)	9 (75.0)	<0.01
Floaters†	3 (5.7)	0	3 (25.0)	0.01

*Values are no. (%), except as indicated. Mann-Whitney-Wilcoxon test was used to compare continuous and the mid-p exact tests for proportions between subgroups. IQR, interquartile range; logMAR, logarithm of the Minimum Angle of Resolution scale; VA, visual acuity. †Statistical significant at α = 5%. **‡For visual acuity baseline, binocular involvement was considered the worst VA.**

**Figure 1:**
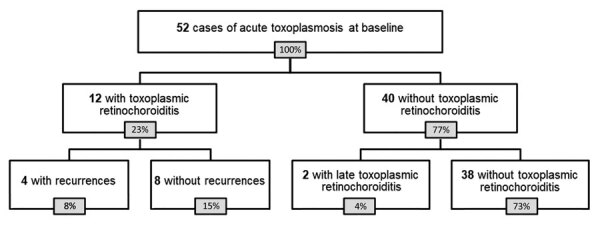
Flowchart of patients in study of ocular involvement associated with a presumed waterborne toxoplasmosis outbreak, Brazil.

At baseline, 12 (23%) patients had retinochoroiditis (Figure 1), 4 (33%) of whom had bilateral involvement and 2 (17%) of whom had macular involvement. Necrotizing lesions or scars were found in 10 (58%) patients; subtle punctate active lesions were found in 4 (33%) patients. Among 4 (33%) patients, we observed multiple necrotizing lesions or multifocal punctate active lesions in different retinal quadrants (Table 2). SLE revealed all 12 patients with toxoplasmic retinochoroiditis had inflammatory cells in the anterior vitreous; however, only 3 (25%) had inflammatory cells in the anterior chamber with standardization of uveitis nomenclature (SUN) grade ranging from 0.5+ to 3+. Intraocular pressure was within normal limits in all but 1 patient with granulomatous keratic precipitates and SUN of 3+ in the anterior chamber. Two (17%) patients with retinochoroiditis did not report any eye symptoms (Table 3). 

**Table 2:** Characterization of patterns of retinochoroiditis seen in multimodal imaging among patients with toxoplasmosis treated with antiparasitic drugs and oral corticosteroids, Brazil*

Type of lesion, fundus imaging modality	Patterns of retinochoroiditis		
	Active phase, before treatment		Cicatricial phase, after treatment
Focal necrotizing retinochoroiditis			
Fundus photo or examination	Dense focal retinal whitening with indistinct borders, associated with overlying vitreous haze		Initially hypopigmented retinochoroidal scar, but frequently evolving with variable degree of pigmentation and subretinal fibrosis or preretinal gliosis
SD-OCT	Focal full-thickness hyper-reflectivity and disorganization of retinal layers indicating necrotizing retinitis; surrounding retinal thickening, signaling edema; numerous overlying hyper-reflective dots at the vitreous indicating vitreal inflammatory cell exudate; and underlying fusiform choroidal thickening, with loss of stromal/luminal pattern indicating reactive choroiditis		Disorganization of retinal architecture; hyper-reflectivity at the level of the scar, but without perilesional retinal thickening; resolution of choroidal thickening; marked decrease in the number of overlying vitreal hyper-reflective dots; and frequent tent-like focal detachment of the less thickened overlying posterior hyaloid
FAF reflectances	Subtle hypo- or hyper-autofluorescence changes at the level of the active lesion; near infrared reflectance can indicate active focus but not as remarkably as red-free reflectance		Increased autofluorescence signal in the first weeks, then hypo-autofluorescence at the level of the scar after several months; scars less clearly delineated by near-infrared than red-free reflectance, but both reveal retinal wrinkling in the presence of epiretinal membrane
FFA	Early hypofluorescence, with progressive hyperfluorescence and late leakage at the retinochoroiditis lesion; reactive changes, including hyperfluorescence, of optic disc indicating edema; staining of venular walls, signaling periphlebitis		Variable window defects and blockage at the level of the scar; staining in the presence of subretinal fibrosis and epiretinal gliosis
Punctate retinochoroiditis			
Fundus photo or examination	Multiple subtle, indistinct, or confluent gray-whitish punctate retinal infiltrates with minimal vitreous haze		Very subtle changes in retinal reflex, sometimes with minor hypopigmentation, but frequently with no apparent abnormality
SD-OCT	Multifocal hyper-reflectivity at the inner retinal layers, demonstrating retinitis, occasionally extending to deeper layers, with surrounding retinal thickening (edema); numerous overlying hyper-reflective dots indicating vitreal inflammatory cell exudate, along with thickening and shallow detachment of the posterior hyaloid; mild choroidal thickening without apparent major change in reflectivity		Frequent normalization of the retinal architecture, sometimes with mild disruption of outer retinal layers or retinal pigment epithelium; normalization of choroidal thickening; marked decrease in the number of overlying vitreal hyper-reflective dots and frequent tent-like focal detachment of the less-thickened overlying posterior hyaloid
FAF reflectances	Subtle hypo- or hyper-autofluorescence changes at the level of the punctate active lesions; near-infrared reflectance can show changes at the area of active foci but not as remarkably as red-free reflectance		Autofluorescence and reflectance changes are minimal or absent
FFA	Progressive but mild hyperfluorescence or late leakage at the site of punctate lesions; reactive changes, including hyperfluorescence of optic disc, demonstrating edema; staining of venular walls indicating periphlebitis		Normal or showing minimal punctate window defects


*FAF, fundus autofluorescence; FFA, fundus fluorescein angiography; SD-OCT, spectral-domain optical coherence tomography.

**Table 3:** Ocular characteristics, recurrences, and complications of patients with ocular involvement from toxoplasmosis, Brazil*

Age, y/sex	Baseline eye examination			Follow-up findings	Complications	Last VA, mo; result
	RC	Right	Left			
38/M†	Bilateral	VA 0.0; SLE, AV cells and AC cells (0.5+/4+); FE, multifocal PR and peripheral large FNR	VA 0.0; SLE, AV cells; FE, PR	1 OD recurrence; month 2, satellite active lesion	None	21; 0.0 OU
47/M†	Bilateral	VA 0.2; SLE, AV cells;FE, peripheral large FNR	VA 0.0; SLE, AV cells; FE, multiple peripheral large FNR	1 OD recurrence; month 22, new peripheral scar	Month 22, epiretinal membrane OD	34; 0.0 OU
40/M†	Bilateral	VA 0.0; SLE, AV cells; FE, multifocal PR	VA 0.0; SLE, AV cells; FE, multifocal PR and peripheral large FNR	Multiple recurrences OU; months 11, 21, and 24, active peripheral lesions; month 27, active peripheral lesion OS	Month 21, epiretinal membrane OD; month 27, rhegmatogenous RD OS	36; 0.0 OD, 0.8 OS
48/M†	Bilateral	VA 2.1; SL, EAV cells; FE, macular FNR	VA 0.3; SLE, AV cells; FE, peripheral FNR	2 recurrences OS; new peripheral scar in months 12 and 15	Month 9, epiretinal membrane OD; month 34, epiretinal membrane OS	34; 1.9 OD, 0.4 OS
43/M†	Unilateral	VA 0.0; SLE, normal; FE, Leber miliary aneurysms	VA 0.7; SLE, fine KP, AC cells 2+/4+, and AV cells; FE, peripheral large FNR	─	None	36; 0.1 OS
27/M	Unilateral	VA 0.0; SLE, AV cells; FE, PR	VA 0.0; normal SLE and FE	─	None	23; 0.0 OD
42/M†	Unilateral	VA 0.5; SLE, granulomatous KP, AC cells (3+/4+), AV cells; IOP, 28 mmHg; FE, peripheral large FNR	VA 0.0; normal SLE and FE	1 recurrence OD; month 9, multiple active peripheral lesions OD	Baseline transient IOP elevation OD, 28mmHg	24; 0.0 OD
31/M	Unilateral	VA 0.0; normal SLE and FE	VA 0.0; SLE OS, AV cells; FE, multiple peripheral FNR	─	None	8; 0.0 OS
50/M†	Unilateral	VA 0.5; SLE, AV cells; FE, peripheral large FNR	VA 0.0; normal SLE and FE	─	Month 6, posterior vitreous detachment OD	37; 0.0 OD
47/F†	Unilateral	VA 1.6; SLE, AV cells; FE, macular FNR	VA 0.0; normal SLE and FE	─	None	37; 1.9 OD
40/M†	Unilateral	VA 0.0; SLE, AV cells; FE, peripheral large FNR	VA 0.0; normal SLE and FE	─	None	37; 0.0 OD
45/M	Unilateral	VA 0.0; SLE, AV cells; FE, PR	VA 0.0; normal SLE and FE	─	None	37; 0.0 OD
15/M‡	NA	VA 0.0; normal SLE and FE	VA 0.0; normal SLE and FE	Late ocular involvement; OD VA 0.1; month 34, new peripheral scar	None	34; 0.1 OD
28/F‡	NA	VA 0.0; normal SLE and FE	VA 0.0; normal SLE and FE	Late ocular involvement; OD VA 0.0; month 37, peripheral FNR	None	39; 0.0 OD

*Age represents age at detection of first ocular lesion or scar. AC cells, grading of anterior chamber cells according to Standardization of Uveitis Nomenclature (SUN) working group (*29*); AV cells, anterior vitreous cells; FE, fundus examination; FNR, focal necrotizing retinochoroiditis, large FNR is >3 disk diameters; IOP, intraocular pressure; KP, keratic precipitates; NA, not applicable; OD, oculus dexter (right eye); OS, oculus sinister (left eye); OU, oculus uterque (both eyes); PR, punctate retinochoroiditis; RC, retinochoroiditis; RD, retinal detachment; SLE, slit-lamp examination; VA, visual acuity (log MAR); –, no recurrence or no new lesion. †Patients with severe ocular involvement, including binocular, macular, or extensive necrotizing retinochoroiditis (>3 disk diameters). ‡Patients with initial normal ophthalmic examination.

We observed other fundus changes among patients with confirmed acute toxoplasmosis but without ocular involvement. One patient had unspecific focal retinal pigment epithelium (RPE) hyperplasia in 1 eye; a patient with nyctalopia had bilateral optic disc pallor, vascular attenuation and RPE changes consistent with retinitis pigmentosa; another patient with severe systemic arterial hypertension had bilateral nerve-fiber layer infarcts. One patient with ocular toxoplasmosis in 1 eye had findings consistent with Leber miliary aneurysms in the contralateral eye.

Among the 12 patients with ocular disease at baseline, 9 (75%) had severe ocular involvement, defined by binocular, macular, or extensive (>3 DD) necrotizing retinochoroiditis. Among 9 patients with severe ocular involvement, the median age was 43 years (IQR 40–47 years); among 3 patients without severe ocular involvement the median age was 31 years (IQR 29–38 years), but the difference was not statistically significant (p = 0.14).

Age, self-reported recent reduction of VA, presence of floaters, and greater reduction in VA were associated with ocular involvement at baseline (Table 1). Among patients with ocular involvement, the median age at infection was 43 years (IQR 40–47 years) vs. 31 years (IQR 22–38 years) for patients without ocular involvement (p = 0.02); 9 (75%) patients with ocular involvement were >40 years of age at infection compared with 6 (15%) patients without ocular involvement (relative risk [RR] = 5.0, 95% CI 2.2–11.2; p<0.01). Among these same subgroups, median logMAR VA at examination was 0.1 (IQR 0.0–0.6) for patients with ocular involvement compared with 0.0 (0.0–0.0) for patients without ocular involvement (p = 0.01).

**Figure 2:**
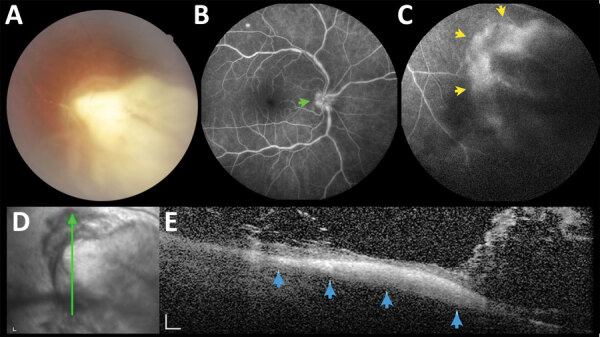
A large necrotizing retinochoroiditis lesion in the right eye, detected in baseline examination (VA 0.5) of a 42-year-old man in a presumed waterborne toxoplasmosis outbreak, Brazil. A) Fundus photograph showing dense focal retinal whitening with indistinct borders, associated with overlying vitreous haze. B) Fundus fluorescein angiography; green arrow indicates hyperfluorescence of optic disc. C) Fundus fluorescein angiography; yellow arrows indicate hyperfluorescence indicating late leakage at the margins of the retinochoroiditis lesion. D) Red-free reflectance showing changes at the level of the active lesion. Green line indicates site of optical coherence tomography scan. E) Spectral-domain optical coherence tomography; blue arrows indicate focal full-thickness hyper-reflectivity and disorganization of retinal layers, surrounding retinal thickening, and numerous overlying hyper-reflective dots and bands, indicating exuberant inflammatory vitreous exudation. Scale bars indicate 200 µm. VA, visual acuity.

**Figure 3:**
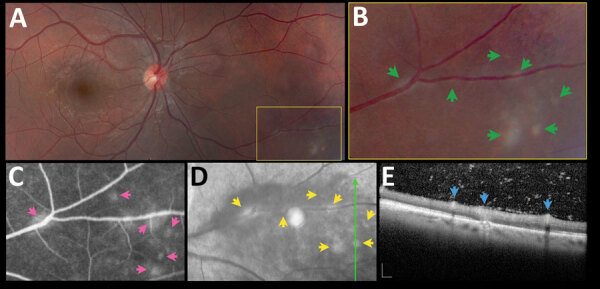
Asymptomatic retinochoroiditis in the right eye, detected in baseline examination (VA 0.0) of a 27-year-old man in a presumed waterborne toxoplasmosis outbreak, Brazil. A) Fundus photograph showing minimal vitreous haze; box indicates enlarged area on B); green arrows indicate multiple subtle and confluent gray-whitish punctate retinal infiltrates. C) Fundus fluorescein angiography. Pink arrows indicate leakage at the site of some of the punctate lesions. D) Fundus fluorescein angiography with red-free reflectance. Green line indicates site of optical coherence tomography scan. Yellow arrows indicate changes in the area of active focuses. E) Spectral-domain optical coherence tomography showing retinal thickening, and numerous overlying hyper-reflective dots. Blue arrows indicate multifocal hyper-reflectivity at the inner retinal layers. Scale bars indicate 200 µm. VA, visual acuity.

Multimodal imaging revealed 2 distinct patterns of active retinochoroiditis: the typical pattern of focal necrotizing retinochoroiditis and punctate retinochoroiditis. Necrotizing retinochoroiditis subsequently left a variably pigmented scar and was more extensive in some cases, simulating a viral retinitis (Figure 2). Punctate retinochoroiditis displayed a much more subtle pattern, which was not seen as easily on fundus examination, but was nicely delineated by SD-OCT (Figure 3). All 12 patients with toxoplasmic retinochoroiditis promptly responded to antiparasitic treatment; 11 received standard therapy with sulfadiazine, pyrimethamine, and folinic acid, supplemented with oral prednisone; 1 had clindamycin instead of sulfadiazine because of sulfa allergy. All 12 patients had resolution of intraocular inflammation within 5–6 weeks. However, the pattern of retinochoroiditis resolution differed between patients with focal necrotizing retinochoroiditis and those with punctate retinochoroiditis (Table 2).

### Follow-up Examinations

Among all 52 patients in the cohort, the median length of follow-up after infection was 36 months (IQR 19–36 months); most (47; 90%) patients were followed for >6 months. Among the 12 patients with ocular involvement at baseline examination, 5 (42%) had recurrent retinochoroiditis during follow-up examinations (Figure 1). The median time for first recurrence was 11 months after starting standard therapy for the first episode of ocular involvement, and the 5 patients had recurrences at 2, 9, 11, 12, and 22 months (Table 3). All 5 reported adequate treatment adhesion. 

**Figure 4:**
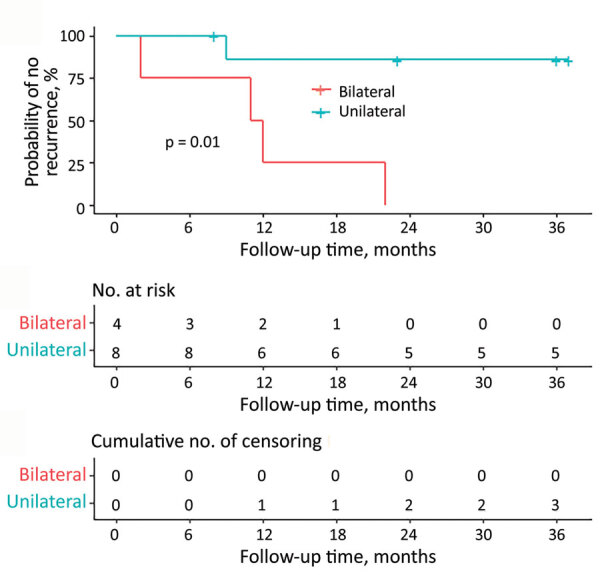
Kaplan-Meier plot showing proportion of patients with toxoplasmic retinochoroiditis at baseline who remain free of recurrence during follow-up. Bilateral retinochoroidal involvement at baseline was statistically significantly associated with recurrences by log-rank test (p = 0.01).

Rate of recurrence of retinochoroiditis among the 12 patients with ocular involvement at baseline was 22% per person-year (1.8% per person-month). All patients with binocular involvement (n = 4) had recurrent lesions during the follow-up period compared with only 13% (1/8) of patients with monocular involvement (RR = 8.0, 95% CI 1.3–50.0; p = 0.01). Rate of recurrence was 8.5% per person-month among patients with binocular involvement and 0.4% per person-month among the patients with monocular involvement (log-rank p = 0.01; Figure 4) (*24*,*25*).

**Figure 5:**
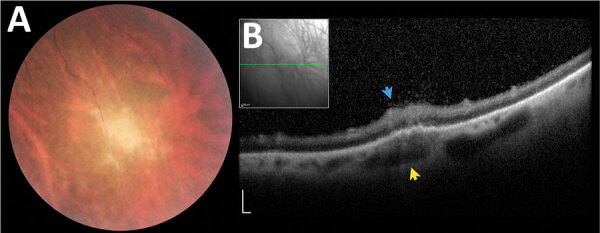
Asymptomatic late retinochoroiditis in right eye detected in follow-up examination at month 37 (visual acuity 0.0) in 28-year-old woman from a presumed waterborne toxoplasmosis outbreak, Brazil. A) Fundus photograph showing focal retinal whitening with indistinct borders. B) Spectral-domain optical coherence tomography showing hyper-reflectivity, disorganization, and thickening of inner retinal layers (blue arrow), and numerous overlying hyper-reflective dots at the overlying vitreous and fusiform thickening of underlying choroid (yellow arrows). Scale bars indicate 200 µm.

Among patients without ocular involvement at baseline, 2/40 (5%) had late ocular involvement. A 15-year-old boy had a new peripheral scar in his right eye (VA 0.1) 34 months after infection, referring a transient VA reduction that started 3 months earlier; the patient recovered spontaneously after a couple of weeks. A 28-year-old woman had a peripheral active lesion in her right eye (VA 0.0) at her 37-month follow-up examination but had no other ocular symptoms (Figure 5).

Overall, 14/52 (27%) patients had ocular involvement at some point during the study. The rate of ocular involvement was 16% per person-year (1.3% per person-month).

### Complications and Visual Outcomes

Among the 16 eyes (15%) of 12 patients (23%) with retinochoroiditis at baseline, ocular complications developed in 7 eyes (44%) of 5 patients (42%) during follow-up; 2 patients had complications in both eyes. Four (25%) eyes had epiretinal membranes develop, detected at 9-, 21-, 22-, and 34-month follow-up visits. One (6%) eye had rhegmatogeneous retinal detachment at the 27-month follow-up, which required pars plana vitrectomy. One (6%) eye had transient intraocular pressure elevation at baseline, and 1 (6%) had posterior vitreous detachment detected at the 6-month follow-up visit.

Among patients with retinochoroiditis at baseline, 4 (25%) eyes among 3 (25%) patients had logMAR VA >0.3 at the last follow-up examination. Two eyes had macular involvement at baseline examination and a final VA of 1.9; an epiretinal membrane developed in 1 eye (final VA 0.4), and rhegmatogeneous retinal detachment developed in the other, which underwent pars plana vitrectomy (final VA 0.8).

## Discussion

We investigated ocular involvement of 52 patients with serologically confirmed acute toxoplasmosis acquired in a presumed waterborne outbreak. We described clinical and multimodal imaging findings and determined the prevalence of retinochoroiditis, the incidence of recurrences and complications, and analyzed risk factors for ocular involvement.

In addition to the standard pattern of necrotizing retinochoroiditis, multimodal imaging revealed a distinct pattern of punctate retinal infiltrates (Figure 3), which might be overlooked if the retina is not examined carefully. This pattern also has been reported in neonates with congenital toxoplasmosis (*5*) and might represent the result of the primary parasite insult to the retina before a more robust immune response develops.

In this study, 12 (23%) patients displayed toxoplasmic retinochoroiditis at baseline examination; 14 (27%) had ocular involvement at some point during the study (Table 3), a rate of 16% per person-year. Variable rates of ocular involvement have been reported in toxoplasmosis outbreaks. For instance, risk for ocular involvement was 31% during the first 10.5 months after the Santa Isabel do Ivaí outbreak in Paraná, Brazil during 2001–2002 (*15*), and 21% after a mean follow-up of 114 weeks in an outbreak in Victoria, British Columbia, Canada in 1995 (*13*,*14*).

As expected, eye symptoms, particularly self-reported recently decreased VA and floaters, were associated with ocular involvement, suggesting that clinicians should inquire about symptoms routinely during and after an outbreak. Nevertheless, 2/12 (16.6%) patients had retinochoroiditis at baseline in the absence of symptoms, reinforcing the importance of examining the eyes of every patient with confirmed acute *T. gondii* infection.

Older age was frequently associated with a higher risk for ocular involvement at baseline and patients >40 years of age at the time of infection had a 5 times greater risk for retinochoroiditis than younger patients. Patients in the subgroup with ocular involvement were much older than patients in the subgroup without ocular involvement, consistent reports in other studies. In a study from the Netherlands, most patients with serologic evidence of recently acquired ocular toxoplasmosis were older, with a mean age of 50.6 years (*26*). A study from Brazil found age was a major risk factor for ocular involvement, with higher prevalence in patients >50 years of age, and »50% of patients >60 years of age had ocular involvement (*10*). Another study from Brazil found that persons >30 years of age with recently acquired *T. gondii* infection were more likely to have ocular involvement by the time of study enrollment (*9*).

At baseline examination, 75% (9/12) of patients with toxoplasmic retinochoroiditis in our study had severe ocular involvement, defined by large (>3 DD) necrotizing, macular, or bilateral retinochoroiditis (Figure 2). The median age of the 9 patients with severe ocular involvement (43 years of age) was higher than the median age of the 3 patients with less severe disease (31 years of age), but the differences were not statistically significant, probably because of the small number of patients in each subgroup. These findings also are in line with the literature (*8*), agreeing with 2 previous studies focusing on ocular toxoplasmosis in patients >50 years of age (median 67.5 years) in which 22/34 (64.7%) patients had severe disease, defined as either multiple active lesions, large lesions (>3 DD), or prolonged disease (duration >8 weeks) (*27*,*28*). Another possible explanation for the higher rate and severity of ocular involvement in our cohort is involvement of a virulent atypical *T. gondii* strain*.* However, this possibility remains elusive because parasite isolation for subsequent genotyping was not successful in the presumably contaminated water source, or in blood samples of patients with serologically confirmed infection (*20*).

Recurrence of retinochoroiditis was associated with bilateral ocular involvement at initial examination (Figure 4). One possible explanation is that patients with bilateral lesions at initial examination might have had a higher parasite load systemically and in the retina, leading to an increased risk for local reactivation. This finding also might have been associated with some degree of subclinical immune dysfunction in these cases.

The most common complication was epiretinal membrane development in 25% of eyes with retinochoroiditis, a finding consistent with a transversal study including 248 patients with acquired toxoplasmosis in India, which also reported development of epiretinal membranes at 25.1% (*12*). At the last follow-up examination 3/12 (25%) patients with retinochoroiditis had logMAR VA >0.3, one of them in both eyes. Complications underlying this visual impairment included unilateral macular involvement at baseline in 2 patients (VA 1.9) and other late complications, including 1 eye with an epiretinal membrane (VA 0.4), and the other with a rhegmatogenous retinal detachment (VA 0.8 after pars plana vitrectomy). 

In addition to the 4 patients who developed recurrences of retinochoroiditis at 2, 9, 11, 12, or even at 22 months of follow-up, 2 patients without retinochoroiditis had new primary retinal lesions detected >34 months of follow-up (Figure 5). This finding highlights the need for long-term ophthalmologic follow-up in patients with postnatally acquired toxoplasmosis, regardless of previous ocular involvement.

This study has some limitations. We only considered symptomatic patients as having suspected cases of acute toxoplasmosis, and that might represent only a small portion of infected persons; several asymptomatic persons probably were not included, and this selection bias might justify, at least in part, the high prevalence of ocular changes. However, selecting for symptomatic cases has been the rule in most investigations of outbreaks of toxoplasmosis because serologic survey of all persons in affected areas is difficult. Finally, incidence calculations were based on few events, especially within subgroups of cases with ocular involvement, and some statistical tests were underpowered, such as the comparison of age in relation to severity of ocular involvement.

Despite the limitations, our study provides objective documentation of ocular changes in the context of a toxoplasmosis outbreak. By using multimodal imaging, we were able to characterize more subtle lesions, such as punctate retinochoroiditis among 33% of cases with ocular involvement. By comparison, previous reports of ophthalmic assessment of patients during outbreaks mostly were based on indirect ophthalmoscopy alone. Among 52 patients with confirmed infection, 47 (90%) had >6 months of follow-up; the median length for follow-up was 36 months, which enabled us to estimate the incidence of new lesions, recurrences, and complications during a relatively long period. We noted a substantial prevalence of early ocular involvement, with recurrences and new lesions occurring up to 39 months after infection. Thus, patients diagnosed with toxoplasmosis should receive long-term ophthalmic follow-up, regardless of initial ocular involvement. In addition, older patients had higher risk for ocular involvement, possibly reflecting age-related changes in the immune system, which could predispose persons >40 years of age to more severe disease. 
